# Prediction of outcomes by diffusion kurtosis imaging in patients with large (≥5 cm) hepatocellular carcinoma after liver resection: A retrospective study

**DOI:** 10.3389/fonc.2022.939358

**Published:** 2022-11-16

**Authors:** Yun-Long Qin, Shuai Wang, Fei Chen, Hong-Xiu Liu, Kui-Tao Yue, Xi-Zhen Wang, Hou-Fa Ning, Peng Dong, Xiang-Rong Yu, Guang-Zhi Wang

**Affiliations:** ^1^ School of Medical Imaging, Weifang Medical University, Weifang, Shandong, China; ^2^ Department of Medical Imaging Center, Affiliated Hospital of Weifang Medical University, Weifang, Shandong, China; ^3^ Department of Radiotherapy, Affiliated Hospital of Weifang Medical University, Weifang, Shandong, China; ^4^ Department of Radiology, Zhuhai People’s Hospital, Zhuhai Hospital affiliated With Jinan, Zhuhai, Guangdong, China

**Keywords:** hepatocellular carcinoma (HCC), diffusion kurtosis imaging (DKI), liver resection (LR), large, outcome, prediction

## Abstract

**Purpose:**

To evaluate preoperative diffusion kurtosis imaging (DKI) in predicting the outcomes of large hepatocellular carcinoma (HCC) after liver resection (LR).

**Materials and methods:**

From January 2015 to December 2017, patients with a large (≥5cm) HCC who underwent preoperative DKI were retrospectively reviewed. The correlations of the mean kurtosis (MK), mean diffusivity (MD), and apparent diffusion coefficient (ADC) with microvascular invasion (MVI) or histological grade were analyzed. Cox regression analyses were performed to identify the predictors of recurrence-free survival (RFS) and overall survival (OS). A nomogram to predict RFS was established. *P*<0.05 was considered as statistically significant.

**Results:**

A total of 97 patients (59 males and 38 females, 56.0 ± 10.9 years) were included in this study. The MK, MD, and ADC values were correlated with MVI or histological grade (*P*<0.01). With a median follow-up time of 41.2 months (range 12-69 months), 67 patients (69.1%) experienced recurrence and 41 patients (42.3%) were still alive. The median RFS and OS periods after LR were 29 and 45 months, respectively. The 1-, 3-, and 5-year RFS and OS rates were 88.7%, 41.2%, and 21.7% and 99.0%, 68.3%, and 25.6%, respectively. MK (*P*<0.001), PVT (*P*<0.001), and ADC (*P*=0.033) were identified as independent predictor factors for RFS. A nomogram including the MK value for RFS showed the best performance, and the C-index was 0.895.

**Conclusion:**

The MK value obtained from DKI is a potential predictive factor for recurrence and poor survival, which could provide valuable information for guiding the efficacy of LR in patients with large HCC.

## Introduction

Hepatocellular carcinoma (HCC) is one of the most common malignant tumors and is ranked as the third leading cause of cancer death worldwide (1). Patients with HCC are usually clinically asymptomatic, and most are diagnosed at an advanced stage or large size. HCC lesions exceeding 5 cm in diameter are defined as large HCCs and account for around 70% of all HCC cases (2). Liver resection (LR) has been considered as the preferred treatment option for solitary large HCC (3). However, patients with large HCC are at an advanced or a late stage and always experience cirrhosis or other complications, which are a challenge for LR. Furthermore, the long-term prognosis after curative LR remains unsatisfactory due to a very high tumor recurrence rate, which results in a median progression free survival (PFS) ranging from 12 to 26 months (4, 5). The optimal classification and management of patients with a large HCC remains a controversial issue, and it is necessary to explore prognosis indicators to guide further treatments.

Previous studies have identified some pathological factors, such as microvascular invasion (MVI), vascular tumor thrombus, histological grading, and tumor size, as independent risk factors for a poor prognosis of HCC (6). However, entire pathological characteristics can only be obtained after resection; non-invasive imaging technologies, such as magnetic resonance imaging (MRI) and computed tomography (CT), provide valuable information for diagnosis and predicting prognosis (7). Traditionally, diffusion-weighted imaging (DWI) derived from MRI is a routine functional imaging method reflecting the diffusion of water molecules obeying a Gaussian distribution in lesions. However, due to the complexity of the internal tissue composition of HCC and the microstructure of tumor cells, the movement and distribution of water molecules show an essentially non-Gaussian distribution (8). Furthermore, diffusion kurtosis imaging (DKI) can potentially be used to explain the non-Gaussian diffusion characteristics of water in complex structures (9, 10). Currently, DKI parameters have been applied in the diagnosis and treatment of solid malignant tumors to improve the characteristics and classification of tumors, such as gliomas and kidney and prostate malignancies (11–14). Previous studies have shown that the DKI of HCC is related to MVI and histological grading (15). However, there have been few studies on the prediction of recurrence and survival after LR in patients with a large HCC by DKI parameters. Thus, we investigated the features of functional parameters derived by DKI in large HCCs and evaluated the efficacy of DKI in prognosis evaluation for LR.

## Materials and methods

### Patients

This retrospective study received approval from the Institutional Review Board (No. wyfy-2022-ky-178, Jan.10, 2022), and informed consent was waived. From January 2015 to December 2017, a total of 175 consecutive patients with a large (≥5cm) HCC who received MRI examinations (including a routine plain scan, a dynamic enhanced scan, DKI, and DWI sequences) before LR were retrospectively reviewed.

**Figure 1:**
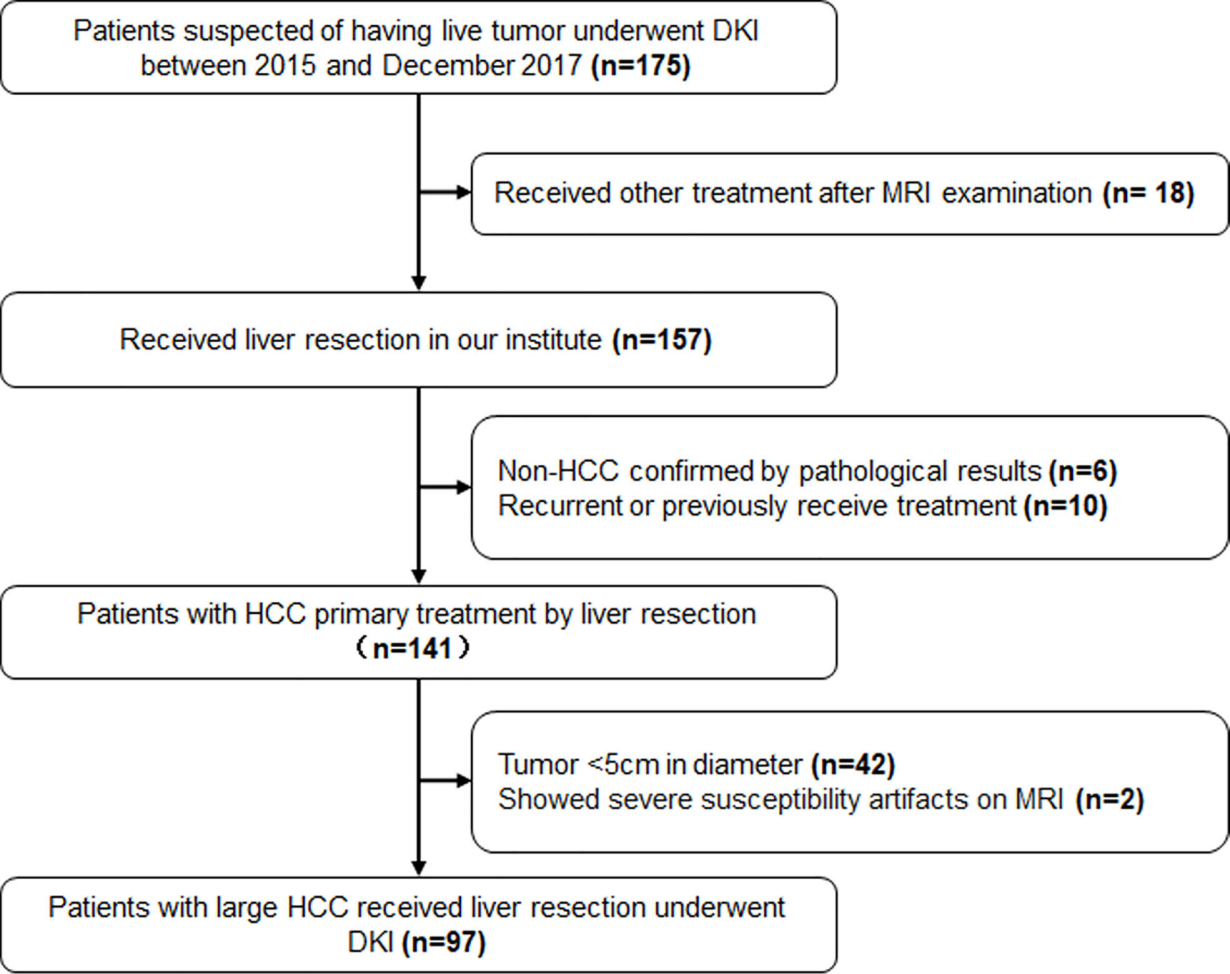
The flowchart of patients’ selection. DKI, Diffusion Kurtosis Imaging; HCC, Hepatocellular Carcinoma.

The exclusion criteria included (1) non-HCC confirmed by biopsy or post-surgical pathological results (2); recurrence or previously received other treatment for HCC (3); tumor <5cm in diameter; and (4) severe artifacts on the MRI that affect image analysis. The patients’ selection was shown in **Figure 1**.

Patient background and laboratory examinations before LR were reviewed for all patients, which included routine blood tests, liver function and alpha fetal protein (AFP) levels, tumor diameter, Eastern Cooperative Oncology Group (ECOG) score, Barcelona Clinic Liver Cancer (BCLC) stage, and Child–Pugh score.

### MRI imaging

MRI was performed using a 3.0 T MR scanner (MAGNETOM Skyra, Siemens Healthcare, Erlangen, Germany) with an 18-channel phased array body coil. All patients fasted for 6-8 hours prior to examination. The MRI sequence that we used was a respiratory-triggered fat-suppressed single-shot echo-planar DKI sequence, and the imaging parameters were as follows: repetition time (TR) = 3300 ms, echo time (TE) = 88 ms, flip angle (FA) = 90°, slice thickness = 5 mm with a slice gap of 1.5 mm, field of view (FOV) = 380×420 mm^2^, matrix size = 168×105, and acquisition time = 5 min. Four b-values of 0, 800, 1500, and 2000 s/mm^2^ were obtained in at least 3 gradient directions. The scan range extended from the top of the diaphragm to the lower end of the liver.

### Image post-processing and analysis

**Figure 2:**
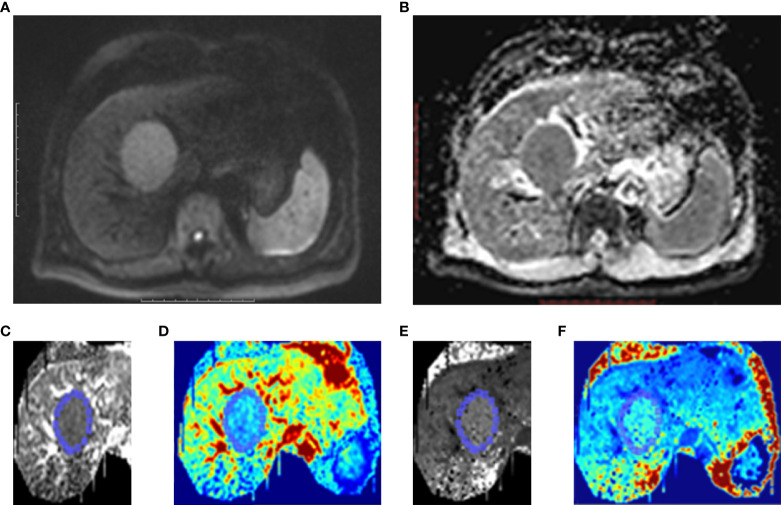
One case of HCC patients without MVI. Male, age 39 years, hepatitis B (+). **(A)** A lesion located at left lobe of liver showed high signal intensity on Diffusion-weighted imaging (DWI). **(B)** On ADC map, the lesion showed as restricted diffusion. Kurtosis map **(C)** and corresponding pseudo-color kurtosis images **(D)** showed lower signal intensity compared with that of liver parenchyma. Diffusion map **(E)** and corresponding pseudo-color diffusion images **(F)** showed iso-intensity compared with that of liver parenchyma.

Publicly available post-processing software (DKE, Medical University of South Carolina, Charleston, USA) was used to generate apparent diffusion coefficient (ADC) and DKI maps. According to the DKI model, S=S_0_·exp(–b·D+b^2^·D^2^·K/6), where b represents the b-value, D represents the corrected apparent diffusion accounting for non-Gaussian diffusion behavior, and K represents the apparent kurtosis coefficient (the deviation of tissue diffusion from a Gaussian distribution). The software also calculated the ADC for pixel size using b-value = 0 and 800 s/mm^2^ based on a mono-exponential model: S =S_0_ ·exp (−b·ADC) (16, 17). Based on these calculations, D, K, and ADC maps were obtained. ROIs were manually drawn at solid parts of the lesions while avoiding large vessels, bile ducts, necrotic tissue, and artifacts, which were measured independently by two experienced abdominal imaging radiologists (with more than 10 years of experience). The ROI was drawn on the largest cross-section of the tumor. If there were multiple lesions, the tumor with the largest diameter was selected as the object of study. Each lesion was measured twice and average values including the mean kurtosis (MK), mean diffusivity (MD), and ADC were calculated (**Figure 2**).

### Histopathological analysis

Histopathological examination was performed for all the surgically resected hepatic specimens. Microvascular invasion (MVI) was defined as tumor within a vascular space lined by endothelium that was visible only on microscopy (18). The HCC histological grade was assigned according to the Edmonson–Steiner system as low grade (grades I and II) or high grade (grades III and IV).

### Follow-up

Contrast-enhanced CT or MRI was performed at 1, 3, 6, and 12 months and then at 6-month intervals after LR to assess tumor response. The primary outcome was recurrence-free survival (RFS),which was defined as the duration from the date of LR to the date of recurrence or metastasis, and secondary outcome was overall survival (OS), which was defined as the time interval from LR to death. All patients were followed up clinically and by phone calls.

### Statistical analysis

Continuous variables were expressed as mean ± standard deviation (SD) or as medians, and categorical data were presented as frequencies and percentages. The reliability of the parameters measured by 2 radiologists was assessed using an intraclass correlation coefficient (ICC) (< 0.40 poor, 0.40–0.59 fair, 0.60–0.74 good, and 0.75–1.00 excellent) (19). Spearman correlation analysis was implemented to determine the degree of correlation between the ADC, MD, and MK values and MVI and histological grade. Receiver operating characteristic (ROC) analyses were depicted to evaluate the corresponding parameters and identify the cutoff values. The Kaplan–Meier survival curves were used for survival analysis. Univariate and multivariate Cox proportional hazards regression analyses were performed to identify the predictors of RFS and OS. Odds ratios (ORs) and 95% confidence intervals (CIs) were calculated. According to the results of the Cox proportional hazards regression analyses, a nomogram to predict RFS was established by the package of rms in R version 4.0.5 (20). MedCalc (MedCalc Software, Ostend, Belgium) were used for statistical analysis. *P *< 0.05 was considered statistically significant.

## Results

### Clinical characteristics of patients

A total of 97 patients (59 males and 38 females, 56.0 ± 10.9 years, range of 32-80) with a large HCC who underwent preoperative DKI were included in this study. There were 67 patients (69.1%) were hepatitis B surface antigen positive, and 5 (5.1%) were hepatitis C infection positive. A total of 61 patients (62.3%) showed AFP levels of ≥400 ng/ml. There were 85 patients (87.6%) with a solitary tumor, and the diameter was 9.91 ± 3.37 cm (range of 5.0-17.8). Pathologically identified MVI was found in 61 patients (62.9%). According to the Edmondson–Steiner classification, 39 cases (40.2%) were classified as low grade, while 58 (59.8%) were classified as high grade. The baselines of clinical and radiological characteristics of all patients are shown in **Table 1**.

**Table 1:** The Baseline Clinical Characteristics of the Patients with Large HCCs.

	Total (N = 97)	Recurrence (–) (n = 30)	Recurrence(+) (n = 67)	*P*
Age	56.0 ± 10.9	56.0 ± 12.1	56.0 ± 10.5	0.994
Gender				0.573
Male	38 (39.2%)	10 (33.3%)	28 (41.8%)	
Female	59 (60.8%)	20 (66.7%)	39 (58.2%)	
Hepatitis virus				0.164
Negative	25 (25.8%)	11 (36.7%)	14(20.9%)	
Positive	72 (74.2%)	19 (63.3%)	53 (79.1%)	
BCLC stage				0.579
A	58 (59.8%)	20 (66.7%)	38 (56.7%)	
B	13 (13.4%)	4 (13.3%)	9 (13.4%)	
C	26 (26.8%)	6 (20.0%)	20 (29.9%)	
Diameter	9.91 ± 3.37			**0.017**
5-10cm	52 (53.6%)	22 (73.3%)	30 (44.8%)	
>10cm	45 (46.4%)	8 (26.7%)	37 (55.2%)	
ECOG PS				0.722
0	71 (73.2%)	23 (76.7%)	48 (71.6%)	
1	20 (20.6%)	6 (20.0%)	14 (20.9%)	
2	6 (6.2%)	1 (3.3%)	5 (7.5%)	
Child-Pugh				0.7
A	72 (74.2%)	21 (70.0%)	51 (76.1%)	
B	25 (25.8%)	9 (30.0%)	16 (23.9%)	
Number				**0.032**
Multiple	12 (12.4%)	0	12 (17.9%)	
Solitary	85 (87.6%)	30 (100%)	55 (82.1%)	
AFP level				0.098
≥400ng/mL	61 (62.9%)	23 (76.7%)	38 (56.7%)	
<400ng/mL	36 (37.1%)	7 (23.3%)	29 (43.3%)	
PVT				**0.005**
Negative	75 (77.3%)	29 (96.7%)	46 (68.7%)	
Positive	22 (22.7%)	1 (3.3%)	21 (31.3%)	

P value < 0.05 showed in bold.

### Features of MK, MD, and ADC in large HCCs

**Figure 3:**
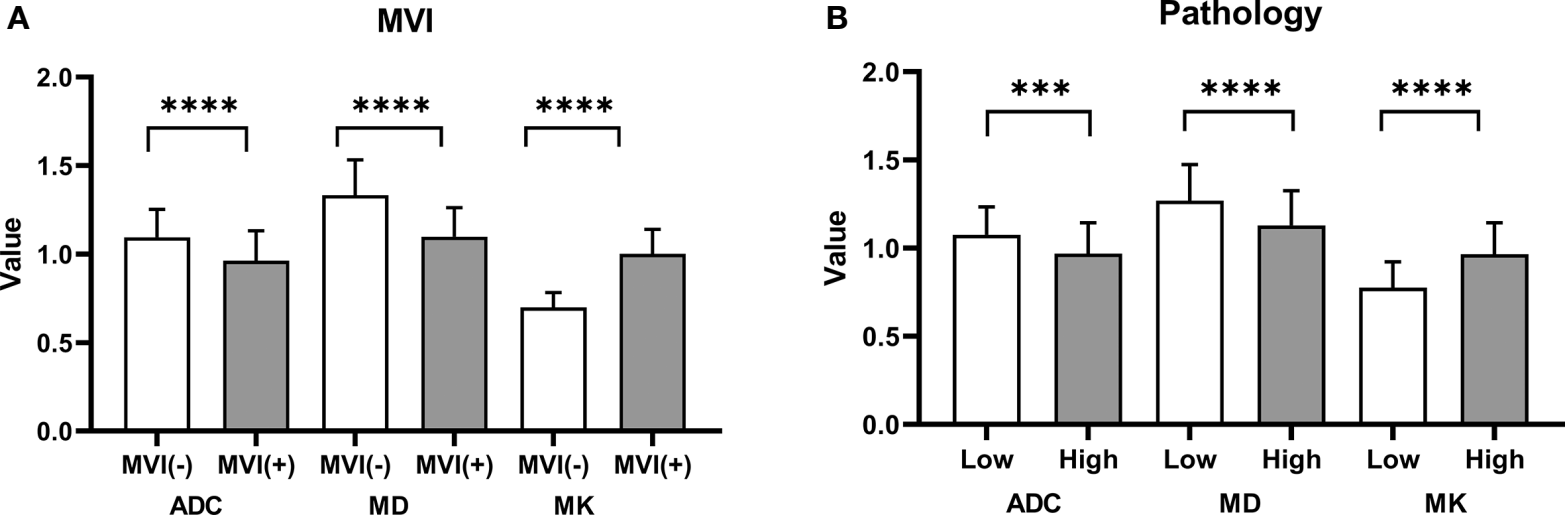
Histogram of the ADC, MK, and MD values classified by MVI and pathology grade. **(A)** Significant differences were observed in the ADC, MK, and MD values between groups with MVI and without MVI. **(B)** The differences in the ADC, MK, and MD values between a high grade and a low grade were statistically significant. ****P < 0.001; ***P < 0.01.

Agreements of the MK, MD, and ADC values between two radiologists were excellent (ICC_MK: 0.912, 95% CI: 0.871-0.940; ICC_MD: 0.822, 95% CI: 0.744-0.877; ICC_ADC: 0.732, 95% CI: 0.624-0.812). The MK values in large HCCs with MVI (1.00 ± 0.14) were higher than those without MVI (0.70 ± 0.08) (*P*<0.001), and the MD and ADC values were lower in large HCCs with MVI than those without MVI (MD: 1.10 ± 0.16 vs. 1.33 ± 0.20, *P*<0.001; ADC: 0.96 ± 0.17 vs. 1.09 ± 0.16, *P*<0.001) (**Figure 3A**). The difference of MK values between high grade and low grade were statistically significant (0.97 ± 0.18 vs. 0.78 ± 0.15, *P*<0.001), while the MD (1.13 ± 0.20 vs. 1.27 ± 0.21, *P*=0.001) and ADC (0.97 ± 0.18 vs. 1.08 ± 0.16, *P*=0.003) values were significantly different between high grade and low grade (**Figure 3B**). The MK values were positively correlated with MVI (rho_MK =0.785, *P*<0.001), while the MD and ADC values were negatively correlated with MVI (rho_MD = -0.530, *P*<0.001; rho_ADC =-0.392, *P*<0.001). In addition, the MK, MD, and ADC values were also correlated with histological grade (rho_MK =0.486, *P*<0.001; rho_MD = -0.291, *P*=0.004; rho_ADC = -0.297, *P*<0.001).

**Figure 4:**
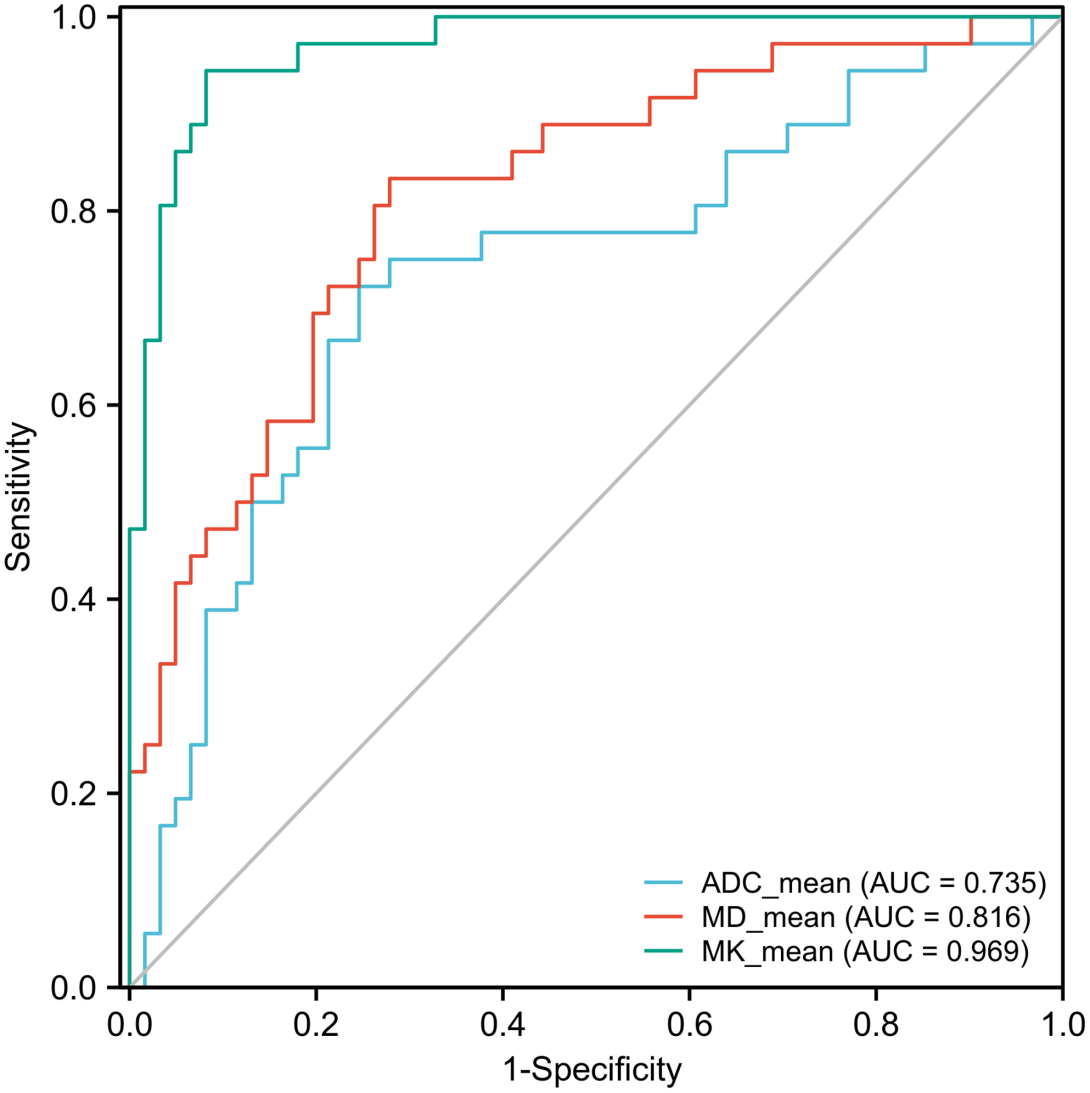
Receiver operating characteristic (ROC) curves of the ADC, MK, and MD for discriminating MVI. The area under the curve (AUC) values of the ADC, MD, and MK were 0.735 (95% CI: 0.635-0.819), 0.816 (95% CI: 0.725-0.888), and 0.969 (95% CI: 0.912-0.994), respectively. The AUCs of the ADC, MD, and MK were statistically significant from each other (*P*<0.001).

The ROC curve showed that MK had a larger area under the curve (AUC) for identifying MVI, with a value of 0.969 (95% CI: 0.912-0.994), than ADC, which had an AUC of 0.735 (95% CI: 0.635-0.819; *P*<0.0001), or MD, which had an AUC of 0.816 (95% CI: 0.725-0.888; *P*=0.0012) (**Figure 4**). The cutoff values of MK, MD, and ADC were 0.810, 1.170, and 1.038 (×10^-3^ mm^2^/sec), respectively. In addition, the Youden index of MK, MD, and the ADC were 0.8625, 0.5546, and 0.4763, respectively (**Table 2**).

**Table 2:** Diagnostic efficacy of MK, MD and ADC in identifying MVI.

	Cut-Off	Sensitivity	Specificity	Youden	AUC	95%CI
MK	0.810	91.8%	94.44%	0.863	0.969	0.912–0.994
MD (×10^-3^ mm^2^/sec)	1.170	72.13%	83.33%	0.555	0.816	0.725–0.888
ADC (×10^-3^ mm^2^/sec)	1.038	75.41%	72.22%	0.476	0.735	0.635–0.819

### Risk factors for outcomes

**Figure 5:**
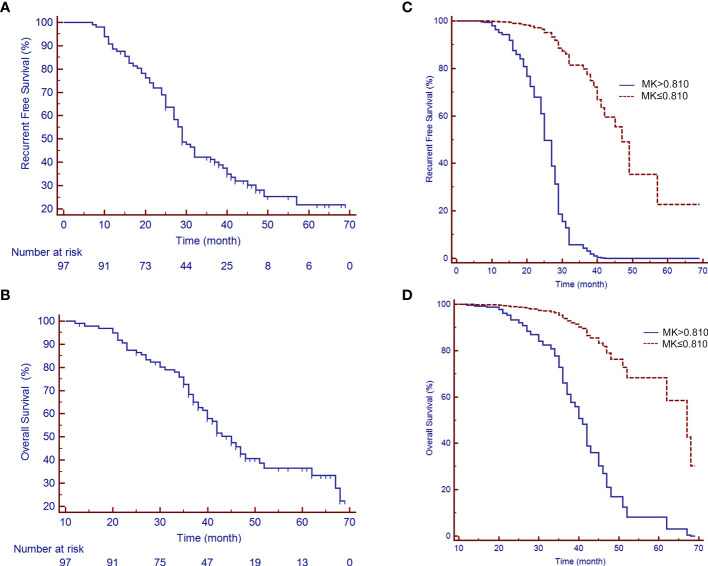
Recurrence-free survival (RFS) and overall survival (OS) rates of patients with a large HCC after liver resection (LR). **(A)** A median RFS period of 29 months (95% CI: 25.88-35.12) and **(B)** a median OS period of 45 months (95% CI: 40.50-49.50) were achieved after LR. **(C, D)** show the survival curves of RFS and OS, respectively, by the MK value after adjusting for potential confounders.

At a median follow-up period of 41.2 months (range 12-69 months), a total of 67 patients (69.1%) had experienced tumor recurrences, 4 patients (4.1%) were lost to follow-up, 1 patient (1.0%) had died from myocardial infarction, and 41 patients (42.3%) were still alive. The difference in the MK, MD, and ADC values between recurrence or not were statistically significant (MK: 0.98 ± 0.15 vs. 0.69 ± 0.08, *P*<0.001; MD: 1.14 ± 0.19 vs. 1.29 ± 0.23, *P*=0.001; ADC: 0.98 ± 0.18 vs. 1.08 ± 0.14, *P*=0.015). The median RFS and OS periods were 29.0 (95% CI: 27.0-37.0) and 45.0 (95% CI: 40.0-51.0) months, respectively (**Figures 5A, B**). The 1-, 3-, and 5-year RFS and OS rates after LR were 88.7%, 41.2%, and 21.7% and 99.0%, 68.3%, and 25.6%, respectively (**Figures 5 C, D**).

Univariate analysis showed that multiple tumors (*P*<0.001), tumor size (*P*<0.001), PVT (*P*<0.001), AFP≥400ng/mL (*P*<0.001), ADC values (*P*<0.001), MD values (*P*<0.001), and MK values (*P*<0.001) were significant predictor factors of RFS for patients with large HCCs. Multiple Cox regression analysis, after adjusting for potential confounders, showed that multiple tumors (*P*<0.001; OR: 5.465; 95% CI: 2.487-12.006), AFP≥400ng/mL (*P*=0.002; OR: 2.384; 95% CI: 1.376-4.132), a PVT (*P*<0.001; OR: 5.382; 95% CI: 2.778-10.426), MK>0.810 (*P*<0.001; OR: 13.758; 95% CI: 5.910-32.026), and the ADC<1.038×10^-3^ mm^2^/sec (*P*=0.033; OR: 0.545; 95% CI: 0.312-0.952) were identified as independent predictors for recurrence (**Table 3**). In addition, univariate analysis for the possible predictive factors of OS, AFP≥400ng/mL (*P*<0.001), tumor number (*P*<0.001), tumor size (*P*<0.001), PVT (*P*<0.001), ADC<1.170×10^-3^ mm^2^/sec (*P*<0.001), MK>0.810 (*P*<0.001), and MD<1.170×10^-3^ mm^2^/sec (*P*<0.001) were significant predictor factors of OS for patients with large HCCs. Multiple Cox regression analysis, after adjusting for potential confounders, demonstrated that multiple tumors (*P*=0.001; OR: 4.060; 95% CI: 1.740-9.474), AFP≥400ng/mL (*P*=0.006; OR: 2.254; 95% CI: 1.225-4.046), MK>0.810 (*P*<0.001; OR: 6.553; 95% CI: 2.354-18.240), PVT (*P*<0.001; OR: 4.965; 95% CI: 2.643-9.326), and MD<1.170×10^-3^ mm^2^/sec (*P*=0.005; OR: 0.373; 95% CI: 0.187-0.743) were identified as independent predictors for large HCCs that underwent LR (**Table 4**).

**Table 3:** Uni- and multi-variate analysis of predictors of recurrence free survival.

Factors	Univariate analysis	*P*	Multivariate analysis	*P*
	OR (95%CI)		OR (95%CI)	
Age	1.004 (0.984-1.025)	0.715		
Gender	0.711 (0.437-1.159)	0.171		
Hepatitis virus	1.745 (0.966-3.150)	0.065		
ECOG PS		0.860		
1 to 0	1.036 (0.567-1.890)	0.909		
2 to 0	1.295 (0.514-3.261)	0.584		
Child-Pugh (A-B)	0.890 (0.504-1.571)	0.688		
Tumor number	9.808 (4.755-20.232)	**< 0.001**	5.465 (2.487-12.006)	**< 0.001**
Tumor size	2.376 (1.460-3.867)	**< 0.001**		
BCLC stage		0.451		
B to A	1.331 (0.642-2.761)	0.442		
C to A	1.380 (0.801-2.377)	0.246		
PVT	9.017 (4.915-16.544)	**< 0.001**	5.382 (2.778-10.426)	**< 0.001**
AFP level	2.568 (1.572-4.194)	**< 0.001**	2.384 (1.376-4.132)	**0.002**
ADC value	0.320 (0.186-0.552)	**< 0.001**	0.545 (0.312-0.952)	**0.033**
MD value	0.336 (0.200-0.566)	**< 0.001**		
MK value	19.353 (8.779-42.664)	**< 0.001**	13.758 (5.910-32.026)	**< 0.001**

P value < 0.05 showed in bold.

**Table 4:** Uni- and multi-variate analysis of prognostic factors associated with overall survival of 97 patients with large hepatocellular carcinoma who underwent liver resection.

Factors	Univariate analysis	*P*	Multivariate analysis	*P*
	OR (95%CI)		OR (95%CI)	
Age	1.003 (0.980-1.027)	0.809		
Gender	0.591 (0.345-1.012)	0.055		
Hepatitis virus	1.891 (0.976-3.663)	0.059		
ECOG PS		0.926		
1 to 0	1.113 (0.582-2.129)	0.746		
2 to 0	1.149 (0.410-3.220)	0.792		
Child Pugh (A-B)	1.092 (0.596-2.001)	0.775		
Tumor number	5.274 (2.642-10.528)	**< 0.001**	4.060 (1.740-9.474)	**0.001**
Tumor size	2.951 (1.700-5.122)	**< 0.001**		
BCLC stage		0.259		
B to A	1.634 (0.747-3.576)	0.219		
C to A	1.522 (0.841-2.754)	0.165		
PVT	8.109 (4.521-14.543)	**< 0.001**	4.965 (2.643-9.326)	**< 0.001**
AFP level	3.049 (1.790-5.194)	**< 0.001**	2.254 (1.225-4.046)	**0.006**
ADC value	0.228 (0.118-0.441)	**< 0.001**		
MD value	0.262 (0.141-0.485)	**< 0.001**	0.373 (0.187-0.743)	**0.005**
MK value	13.962 (5.504-35.417)	**< 0.001**	6.553 (2.354-18.240)	**< 0.001**

P value < 0.05 showed in bold.

**Figure 6:**
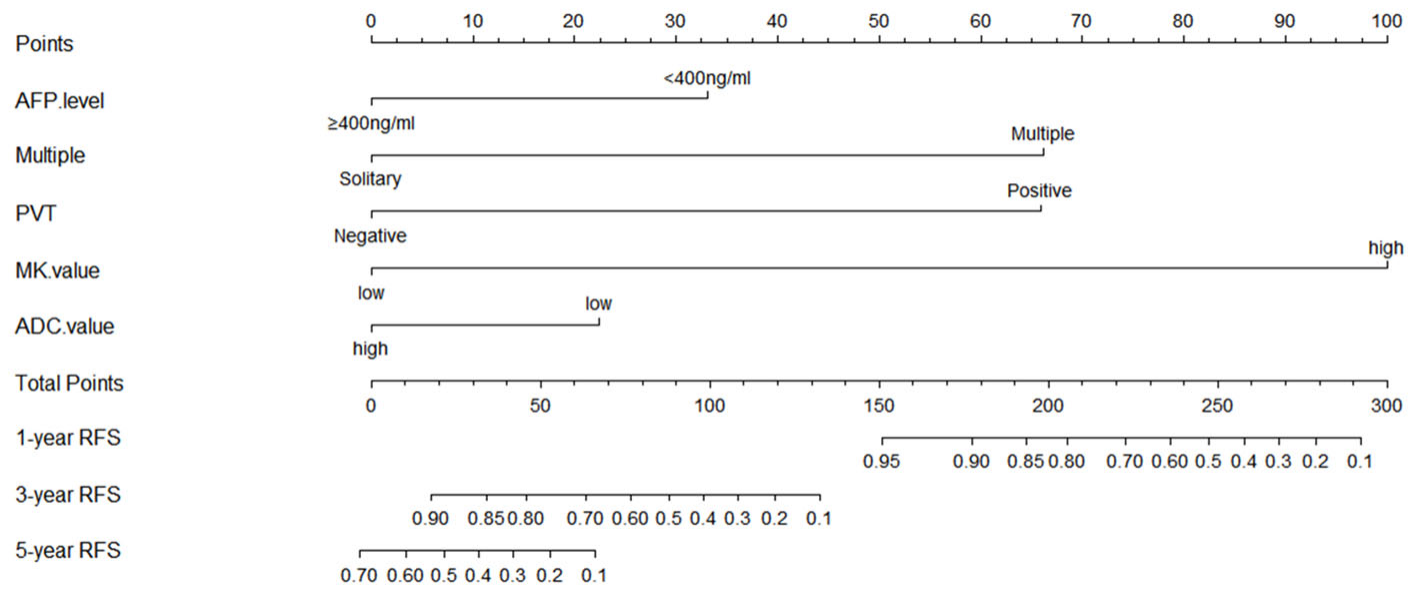
Prognostic nomogram graph for recurrence-free survival. PVT, portal vein tumor; RFS, recurrence-free survival.

A prediction model for RFS was derived on the basis of multivariate Cox regression analysis. A nomogram was constructed on the basis of this prediction model (**Figure 6**). The C-index for RFS prediction was 0.895 (95% CI: 0.88-0.91).

## Discussion

In this study, the DKI parameters were correlated with malignant pathological features, including MVI and degrees of differentiation, and they can predict tumor recurrence and poor survival for patients with large HCCs after resection. Many studies have reported that early recurrence mainly originates from occult tumor lesions that were not identified in pre-/intra-resection; MVI is an important factor in early recurrence after LR (21). The existence of MVI introduces a more complex tumor microenvironment, which limits the movement of water molecules; on the other hand, the proliferation of tumor cells will further change the anatomical structure, causing inflammation, bleeding, and necrosis, thus increasing the complexity of tissue at the microstructure level. Therefore, the ADC values derived from DWI are less effective in evaluating the biological characteristics of heterogeneous HCC, even though they are associated with degrees of differentiation or prognosis (22–25). Nevertheless, the MK values derived from DKI could more sensitively and truly reflect the degree of differentiation and pathologic behavior of the tumor, which depends on the complexity of the tissue structure with significantly more restricted non-Gaussian diffusion in the tumor (15, 16).

In this study, the MK values of patients with MVI were significantly higher than patients without MVI (*P*<0.001), while MD and ADC were on the contrary (*P*<0.001). The ROC showed that sensitivity for the identification of MVI was higher for MK than for MD and ADC. Wang et al. confirmed that DKI has higher value in predicting the existence of MVI than the traditional ADC (17). Nevertheless, neither MD nor ADC was associated with MVI in the study. Cao et al. also reported that MK was effective for predicting MVI, furthermore, they identified MK value was an independent risk factor for recurrence within a year (15). Beyond that, we still further studied DKI in evaluating short- and long-term outcome. Another study also showed that MK provided higher accuracy than ADC in evaluating HCC viability after local treatments (8). Although there were some differences from previous studies, all evidence showed that there is a good correlation between DKI and MVI, which may explain the prognostic effect of DKI.

Although some progress has been made in the treatment of large HCCs (26), LR remains the best potential curative treatment option for patients with large HCC. Previous studies have identified several prognostic factors that affect recurrence and long-term survival in patients with large HCCs after LR, which is critical to patient selection for receiving LR (7). In this study, the MK value (>0.810) was a strong and independent predictor of RFS and OS in patients with large HCCs. Although the ADC value (<1.038×10^-3^mm^2^/sec) was an independent predictor for early recurrence in patients with large HCC, the results were not statistically significant in predicting long-term prognosis, which was in keeping with the studies concerning ADC for the evaluation of MVI in HCC (27). Furthermore, the MD value (<1.170×10^-3^ mm^2^/sec) was found to be an independent predictive factor for OS, but it had limited value in evaluating RFS in this study.

Our study also demonstrated that PVT, high AFP levels (AFP≥400ng/ml), and multiple tumors were correlated with PFS and OS for patients with large HCCs who underwent LR. In addition, Delis et al. (28) reported that tumor size is an independent predictor for recurrence and long-term survival in patients with large HCCs. However, in our study, tumor size did not significantly predict RFS or OS by multiple Cox regression analysis. This may be related to patient selection or pathological grade.

There are various options for the treatment of large HCCs, which vary in different regions (29, 30). With careful patient selection and hepatectomy techniques (11–13, 23, 31), a more safe and potentially curative resection can be performed in many such cases of large HCC. In previous studies, the 1-, 3-, and 5-year OS rates after LR were 68.5%-69%, 37%-47.6%, and 32%-41.3%, respectively (26, 28). However, the 1-, 3-, and 5-year OS rates after LR were 99.0%, 68.3%, and 25.6% in this study. The short-term outcome seems slightly better than other studies, and the 1-, 3-, and 5-year RFS rates after LR were 88.7%, 41.2%, and 21.7%, which could be caused by the strict selection or the conservative concept for patients with a large HCC receiving LR. However, the long-term outcome corresponded to previous studies. As is known to all, the OS can be affected by many factors, such as further treatments after recurrence. Therefore, we mainly investigated the prognostic factors that can affect recurrence. Because of recurrence after LR significantly aggravates long-term survival, the relationship between MK value and OS could be explained based on RFS analysis results. There were many models for predicting prognosis clinically, including an artificial neural network model. However, a nomogram based on the Cox regression model has high accuracy and good discrimination characteristics in predicting outcomes and is easy to use. In the present study, the proposed nomogram for predicting RFS, which incorporated five preoperative clinical and imaging features, performed well, as supported by the C-index value of 0.895. Based on these preoperative indicators (MK value >0.810, ADC value <1.038×10^-3^mm^2^/sec, PVT, multiple tumors, and AFP≥400ng/ml), the nomogram might serve as a tool to select patients for evaluating the efficacy of LR in patients with a large HCC.

Our study had some limitations. First, the sample size of the multivariate analysis was relatively small, which reviewed only cases with a large HCC, and larger samples need to be collected to verify the results. Additional independent external validation sets were lacking, and this will be our important study in the future. Secondly, the measurement of DKI was strongly influenced by the ROI selection. Wei et al. (32) reported that different ROI positioning methods substantially influence the ADC value measurement. In addition, the parameters of DKI can easily be affected by necrosis, so we selected the ROI by avoiding necrotic areas, and the consistency between different evaluations was good. Third, the setting of the b-value is important for DKI. For the correct setting of the b-value and other imaging parameters, there is no standardized scheme in clinical practice at present. Therefore, the use of DKI in prognosis evaluation for large HCCs needs further study.

## Conclusion

In conclusion, the MK value was significantly correlated with MVI and pathological grade in patients with large HCC. A higher MK increases the risk of tumor recurrence and poor survival. Therefore, the DKI technique can provide an excellent reference for evaluating the efficacy of LR in patients with large HCCs.

## Data availability statement

The original contributions presented in the study are included in the article. Further inquiries can be directed to the corresponding authors.

## Ethics statement

The studies involving human participants were reviewed and approved by Institutional Review Board of Weifang Medical University Affiliated Hospital. Written informed consent for participation was not required for this study in accordance with the national legislation and the institutional requirements.

## Author contributions

G-ZW and X-RY participated in the design of the study. Y-LQ and SW wrote the manuscript. FC, H-XL, and K-TY collected and analyzed the data. X-ZW, H-FN, and PD contributed to interpretation of data and preparation of the manuscript. All authors contributed to the article and approved the submitted version.

## Funding

This work was supported in part by grants from the Shandong Province Science and Technology Development Project (No. 2019WS596); and the Shandong Provincial Natural Science Foundation (No. ZR2020MH293 & No. ZR2020MH242).

## Conflict of interest

The authors declare that the research was conducted in the absence of any commercial or financial relationships that could be construed as a potential conflict of interest.

## Publisher’s note

All claims expressed in this article are solely those of the authors and do not necessarily represent those of their affiliated organizations, or those of the publisher, the editors and the reviewers. Any product that may be evaluated in this article, or claim that may be made by its manufacturer, is not guaranteed or endorsed by the publisher.
